# Gut Bifidobacteria Populations in Human Health and Aging

**DOI:** 10.3389/fmicb.2016.01204

**Published:** 2016-08-19

**Authors:** Silvia Arboleya, Claire Watkins, Catherine Stanton, R. Paul Ross

**Affiliations:** ^1^APC Microbiome Institute, University College CorkCork, Ireland; ^2^Teagasc Food Research Centre, Moorepark, FermoyCork, Ireland; ^3^School of Microbiology, University College CorkCork, Ireland; ^4^School of Science, Engineering and Food Science, University College CorkCork, Ireland

**Keywords:** bifidobacteria, intestinal microbiota, health, aging, probiotics

## Abstract

The intestinal microbiota has increasingly been shown to have a vital role in various aspects of human health. Indeed, several studies have linked alterations in the gut microbiota with the development of different diseases. Among the vast gut bacterial community, *Bifidobacterium* is a genus which dominates the intestine of healthy breast-fed infants whereas in adulthood the levels are lower but relatively stable. The presence of different species of bifidobacteria changes with age, from childhood to old age. *Bifidobacterium longum*, *B. breve*, and *B. bifidum* are generally dominant in infants, whereas *B. catenulatum*, *B. adolescentis* and, as well as *B. longum* are more prevalent in adults. Increasingly, evidence is accumulating which shows beneficial effects of supplementation with bifidobacteria for the improvement of human health conditions ranging from protection against infection to different extra- and intra-intestinal positive effects. Moreover, bifidobacteria have been associated with the production of a number of potentially health promoting metabolites including short chain fatty acids, conjugated linoleic acid and bacteriocins. The aim of this mini-review is to describe the bifidobacteria compositional changes associated with different stages in life, highlighting their beneficial role, as well as their presence or absence in many disease states.

## Introduction

The study of the intestinal microbiota, linked with its impact on human health, has become a transforming topic in microbiology research. From the pioneering studies using culture-dependent techniques to the current omics’ approach, composition and functionality of the intestinal microbiota has been assessed in a wide variety of studies (Lagier et al., 2015). The complex microbiota and the relationships it plays in the prevention and alleviation of diseases have been a major focus of many works (Turnbaugh et al., 2009; Qin et al., 2010; Karlsson et al., 2013). Members of the genus *Bifidobacterium* have been identified as almost ubiquitous inhabitants of the human host (Biavatti and Mattarelli, 2006), performing an important role in the gut from birth. Due to the traditional interest in bifidobacteria as probiotics this genus has been studied in depth, and changes in the species composition, diversity or relative abundance have been investigated at different stages of life and in several diseases. The aim of this mini-review is to describe the bifidobacteria compositional changes associated with different stages in life, highlighting their beneficial role, as well as the possible role of their presence in the protection against many disease states.

## Intestinal Microbiota

The intestinal microbiota plays an important role in human health and has long been associated with such functions as metabolic, protective, and trophic (Guarner and Malagelada, 2003) and more recently functions related to the gut-brain axis or liver-gut axis (Clarke et al., 2014). The current exponential increase in sequencing and the explosion of other “omics” approaches has allowed us to gain a deep knowledge of intestinal microbiota.

The establishment of the intestinal microbiota was initially considered to occur at birth; however, the presence of microorganisms in placenta or amniotic fluid (Collado et al., 2016) suggests a primary fetal colonization. Moreover, the process is determined by different factors which undoubtedly have an effect on microbiota homeostasis (Penders et al., 2006; Faa et al., 2013). Actinobacteria, followed by Proteobacteria and Firmicutes are the main phyla in early childhood, characterized by low diversity and complexity (Koenig et al., 2011; Turroni et al., 2012). The main changes in gut microbiota composition take place in the first stages of life, getting to a relative stability at 1–2 years old (Voreades et al., 2014). The adult-like structure of the gut microbiota is thought to occur after the 3rd year of life (Koenig et al., 2011; Yatsunenko et al., 2012), and reaches a total number of 10^14^ microorganisms, comprising of bacteria, eukarya, viruses and archaeal members (Mihajlovski et al., 2010; HMP, 2012). At phylum level, the gut microbiota is made up of 80–90% Firmicutes and Bacteroidetes. At species and strain taxonomic level, the diversity is very high in adult life, and is characterized by high interindividual variability (HMP, 2012). However, the functionality and metabolism of the gut microbiota is highly conserved (Qin et al., 2010). Aging was defined by Imahori (1992) as “the regression of physiological function accompanied by advancement of age.” It is a natural process which entails changes in the gastrointestinal tract (GIT), immunosenescence and, in some cases, malnutrition (Woodmansey, 2007; Biagi et al., 2012). In addition, alterations in lifestyle, diet and medication have an unavoidable effect on the elderly microbiota composition and function (Biagi et al., 2012; Claesson et al., 2012). Microbial diversity is reduced in old age; however, Bacteroidetes and Firmicutes still constitute the dominant phyla, with increases in the relative abundance of some other Phyla – most notably Proteobacteria (Biagi et al., 2012; Odamaki et al., 2016).

## Bifidobacteria And Their Relevance: From The Infant-Type To The Aged-Type Microbiota

**FIGURE 1:**
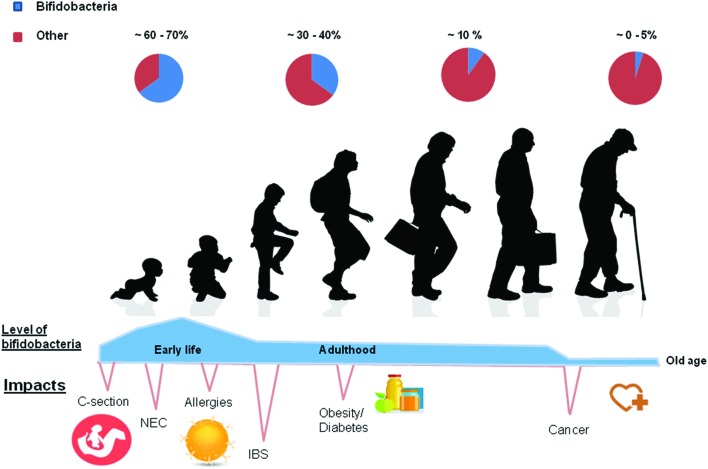
**At birth levels of bifidobacteria are found to be at their highest.** In cases of natural childbirth the numbers are highest at birth. In contrast, they are lower in C sectioned babies. Various diseases such as obesity, diabetes and allergies have been associated with lower numbers of bifidobacteria at various stages of life. When weaned onto solid foods diet is more of an intervening factor and an adult-like (stable) microbiota develops. In this figure the authors hypothesize with regard to the relative abundance of bifidobacteria present at each stage of the life cycle, based on the literature cited in the following review by Voreades et al. (2014).

Bifidobacteria are normal inhabitants of the GIT belonging to the Actinobacteria phylum. After the depletion of oxygen by facultative anaerobes, bifidobacterial populations are the most abundant genus present in the healthy infant gut (Favier et al., 2002). During adulthood the levels decrease considerably but remain relatively stable; decreasing again in old age (Odamaki et al., 2016; **Figure 1**). Certain strains of *Bifidobacterium* are widely used as probiotics – the safety of which is supported by the long historical consumption in fermented milks and the growing knowledge about their physiology and genomes (Arboleya et al., 2016; O’Callaghan and van Sinderen, 2016). The genus *Bifidobacterium* has been shown to play an important role in the barrier effect, the stimulation of immune system, being associated with a range of beneficial health effects (Picard et al., 2005).

### Early Stages of Life

The initial colonization by bifidobacteria is dependent on a number of extrinsic factors. In terms of vertical transfer, many studies have linked the transmission of bifidobacteria from the mother’s vaginal tract, GIT, breast milk, placenta and amniotic fluid to the infant (Makino et al., 2013; Collado et al., 2016). Birth mode, in particular, has a significant impact on this initial colonization, with an increased abundance of bifidobacteria found in infants born vaginally, versus those born by cesarean section (Dominguez-Bello et al., 2010).

Gestational age has been described in terms of its impact on the infant gut, whereby pre-term infants have been characterized in many studies by a dominance of Proteobacteria, with increases in members of *Clostridium* and *Staphylococcus*, and much lower levels of Actinobacteria. In contrast, the full-term infant gut has been correlated with much higher levels of *Bifidobacterium* and *Bacteroides*, which tend to be dominant in the early weeks of life (Arboleya et al., 2012; Barrett et al., 2013; Arboleya et al., 2015).

Numerous studies investigating the effects of breast feeding versus formula feeding have identified specific bifidobacterial species that correlate with the feeding regime (Roger et al., 2010). Using both culture-dependent and molecular methods, studies have found that *Bifidobacterium breve*, *B. bifidum*, *B. longum* ssp. *longum*, and *B. longum* spp. *infantis* are present in both breast- and formula-fed infants (Mevissen-Verhage et al., 1987; Klaassens et al., 2009). However, *B. longum* ssp. *infantis* is more commonly associated with breast fed infants, whereas *B. longum* ssp. *longum* has been found more commonly amongst bottle-fed infants (Guaraldi and Salvatori, 2012). *B. adolescentis*, which is commonly found in adults, was found to be present only in formula-fed babies (Klaassens et al., 2009). Numerous studies have focused on the bifidogenic effect that human breast milk has on the infant gut microbiota (Musilova et al., 2014). Specific glycans found within breast milk are known to be utilized by bifidobacteria; however, it has been shown that breast milk provided by mothers with an inactive allele of the Fucosyltransferase 2 gene (FUT2), an enzyme involved in the transfer of fucose to glycans, delays the establishment of bifidobacteria in the infant (Lewis et al., 2015). Similarly, bifidobacteria provide an important role in the breakdown of human milk oligosaccharides (HMOs; Garrido et al., 2013), creating a clear evolutionary link between the mother, infant and the microbial species present. Indeed, *B. longum* ssp. *infantis* has specifically been studied for its ability to digest different HMO structures (Sela et al., 2008). This species is also associated with anti-inflammatory properties, and the ability to decrease intestinal permeability (Underwood et al., 2015). The growth of infant-derived *Bifidobacterium*, *B. longum* ssp. *infantis*, and *B. bifidum*, in the presence of HMOs has also been shown to promote the adhesive properties of these strains, as well as the expression of anti-inflammatory cytokines and tight junction proteins in eukaryotic cells (Chichlowski et al., 2012). Interestingly, a study published by Del Chierico et al. (2015) focused on the co-correlation between the metabolome and the OTUs occurrence in infant stool samples. Results found that *Bifidobacterium* OTUs were positively correlated with the presence of lactate and alanine during this 1st month of life. Another recent work, negatively correlated the lactase gene with *Bifidobacterium* levels in the gut microbiota of twins, speculating that lactase-persisters babies harbor lower levels of bifidobacteria because of low lactose availability (Goodrich et al., 2016).

In terms of species, *B. longum, B. breve*, and *B. bifidum* are most abundant (**Table 1**) in infants (Favier et al., 2002; Gueimonde et al., 2007; Turroni et al., 2012). A recent study described the different *Bifidobacterium* species profile between monozygotic twins (entirely dominated by *B. breve*) and their fraternal sibling (exhibiting higher species diversity) at 1 month of life (Murphy et al., 2015; **Table 1**).

**Table 1:** **(A)** Distribution of the most abundant *Bifidobacterium* species in the intestinal microbiota at different stages of life analyzed using different techniques.

(A) Human population	*Bifidobacterium* spp.	Techniques	Reference
**Infants**			
Breast-fed, 22–24 days of age	*B. breve^a^* *B. longum* ssp. *longum, B. longum* ssp. *infantis^b^*	PCR	Matsuki et al., 1999
Breast- and Formula fed, 28–90 days of age	*B. longum* ssp. *infantis* *B. breve, B. longum ssp. longum*	PCR	Haarman and Knol, 2005
Breast-fed, 1 month of age	*B. longum* *B. bifidum, B. animalis, B. breve*	PCR	Grönlund et al., 2007
Breast-fed, 3–6 weeks of age	*B. breve* *B. longum ssp. *longum, B. longum* ssp. *infantis**	PCR	Mikami et al., 2009
Full-term, 1 month of age	*B. longum*	q-PCR	Grzeskowiak et al., 2015
Preterm, CS, 1 month of age	*B. longum, B. lactis*		
Preterm, Vaginal, 1 month of age	*B. longum, B. bifidum*		
Twins, 1 month of age	*B. breve*	16S Metagenomics	Murphy et al., 2015
Fraternal infant, 1 month of age	*B. breve, B. longum* *B. dentium, B. adolescentis*		
**Adults**			
23–54 years old, Japanese	*B. catenulatum,* *B. longum, B. adolescentis*	PCR	Matsuki et al., 1999
25–59 years old, Japanese	*B. longum,* *B. adolescentis, B. catenulatum*	q-PCR	Matsuki et al., 2004
≤57 years old, Russian	*B. adolescentis*	MALDI-TOF	Chaplin et al., 2015
20–40 years old, Finnish	*B. longum,* *B. catenulatum*	q-PCR	Gueimonde et al., 2007
18–39 years old, lean subjects (BMI = 19.83 ± 0.94 kg/m^2^)	*B. longum*	q-PCR	Mayorga Reyes et al., 2016
**Elderly**			
69–89 years old, French	*B. adolescentis* *B. longum*	DNA–DNA hybridization	Gavini et al., 2001
67–75 years old, Scottish	*B. angulatum* *B. longum*	Culture-based analyses	Woodmansey et al., 2004
>70 years old, Finish	*B. catenulatum* *B. longum, B. bifidum*	q-PCR	Gueimonde et al., 2007
77–95 years old, Spanish	*B. longum* *B. bifidum, B. pseudocatenulatum*	q-PCR	Salazar et al., 2013
**Centenaries**			
100–104 years old, Italian	*B. longum* *B. adolescentis, B. bifidum*	Culture-based analyses	Drago et al., 2012
80–108 years old, Chinese	*B. dentium* *B. longum*	q-PCR	Wang et al., 2015
**(B) Disease in human population**	***Bifidobacterium spp.***	**Techniques**	**Reference**
**Infants**			
Allergic mothers	↑ *B. adolescentis*	q-PCR	Grönlund et al., 2007
Coeliac disease (Non-active)	↓*B. longum;* ↑ *B. dentium*	q-PCR	Collado et al., 2008a
Celiac disease	↓*B. longum*	q-PCR	Palma et al., 2012
Allergic diseases	↓*B. longum* ↑ *B. pseudocatenulatum, B. catenulatum*	Culture-based analyses	Akay et al., 2014
**Adults**			
Allergy	↑ *B. adolescentis*	PCR-DGGE	Stsepetova et al., 2007
IBS	↓*B. catenulatum*	q-PCR	Kerckhoffs et al., 2009
IBS	↓*B. catenulatum/pseudocatenulatum*	q-PCR	Lyra et al., 2009
IBS	↓*B. pseudocatenulatum, B. gallicum*	HITChip phylogenetic microarray	Rajilic-Stojanovic et al., 2009
IBS	↑ *B. adolescentis*	16S Metagenomics	Jeffery et al., 2012
Hepatitis B virus-related cirrhosis	↑ *B. dentium* ↓*B. catenulatum, B. longum*	PCR-DGGE and q-PCR	Xu et al., 2012
Obesity	↓*B. animalis*	q-PCR	Million et al., 2013
Cystic fibrosis	↓*B. catenulatum/pseudocatenulatum, B. longum, B. adolescentis*	PCR-DGGE	Duytschaever et al., 2013
Long-term asthma	↑ *B. adolescentis*	16S Metagenomics	Hevia et al., 2016

*First row indicates the most abundant species found^a^. Second row indicates other abundant species found^b^. **(B)** Studies focusing on Bifidobacterium species altered in certain diseases.*

↑ *Increased levels*; ↓ *Decreased levels*.

### Adulthood and Old Age

In adulthood, the levels of bifidobacteria are lower (2–14% relative abundance) but remain stable (Odamaki et al., 2016). Gueimonde et al. (2007) identified significantly higher levels of *Bifidobacterium* in infants than in adults by q-PCR technique. They postulated that *B. longum* is the most widely abundant species, which is in agreement with other studies (Gavini et al., 2001; Matsuki et al., 2004). However, Matsuki et al. (2004) observed higher levels of *B. adolescentis* and *B. catenulatum* in their adult population. Chaplin et al. (2015) reported a decreased frequency of isolation of *B. bifidum* and *B. breve* with age and an increased trend in *B. adolescentis*.

Currently, there is no agreed definition of old-age-specific gut microbiota profile due to the high inter-individual variability, differences in diet and lifestyle, and the unclear definition of the term “elderly.” However, some trends are repetitively observed such as the decrease of bifidobacteria in the elderly population, confirmed by several studies using different technologies (Mitsuoka et al., 1974; Mitsuoka, 1992; Gavini et al., 2001; Hopkins et al., 2001; Gueimonde et al., 2010; Biagi et al., 2012; Salazar et al., 2013). Woodmansey et al. (2004) reported that the decline in bifidobacteria population with aging was accompanied by a decrease in species diversity. This decline was associated with the reduction in adhesion to the intestinal mucosa, but it is not clear if it is due to the changes in the microbiota or in the structure of mucus (He et al., 2001).

The bifidobacteria composition in centenary populations was also reported in some studies; however, the results remain somewhat controversial. In a European population, the microbiota composition of centenarians was still similar to that of adults (Biagi et al., 2010), however, higher proportions of bifidobacteria were found in centenarians than in younger elderlies from a region of China (Zhao et al., 2011). Regarding species (**Table 1**), *B. longum* was the most abundant in Italian centenarians followed by *B. adolescentis* and *B. bifidum* (Drago et al., 2012), but *B. dentium* was dominant in Chinese centenaries (Wang et al., 2015).

Other extrinsic factors indirectly related to the aging process also affect the bifidobacteria composition. The extended use of antibiotics in the older population undoubtedly has a huge impact on the intestinal microbiota composition, decreasing the bifidobacteria population (Woodmansey et al., 2004; O’Sullivan et al., 2013). While antibiotics remain an essential medical tool, therapies targeted toward the reestablishment of microbiota have been explored, in particular the use of probiotics to correct the imbalance in the bifidobacteria population and the alteration in the intestinal microbiota after antibiotic therapy (Rondanelli et al., 2015). In terms of frailty, van Tongeren et al. (2005) did not find differences in bifidobacteria between elderly people divided into a low and high frailty category. The same trend was observed by Bartosch et al. (2004) between elderly living in the local community and elderly in a hospitalized environment.

## Bifidobacteria And Diseases

Several diseases, both intra- and extra-intestinal, have been associated with alterations in the gut microbiota composition and function (Wu and Lewis, 2013). Although, there is still not a detailed description of “potential alterated-microbiota types,” some authors postulate that intestinal microbial alterations could be the prelude to a wide range of disease (Putignani et al., 2014).

### Bifidobacterial Composition in Diseases

Given the widespread use of bifidobacteria as probiotics, they have been studied extensively and as such the aberrancies in bifidobacteria species composition, diversity or changes in their relative abundance have been reported in several diseases.

Obesity is a worldwide disease affecting children and adults, which is commonly associated with alterations in the microbiota. Some studies have shown lower levels of bifidobacteria, linked to higher prevalence of enterobacteria or *Staphylococcus* in obese children (Kalliomäki et al., 2008; Gao et al., 2015). Interestingly, women who gain weight during pregnancy have displayed lower levels of *Bifidobacterium* in contrast to healthy weight pregnant women (8.36 vs. 9.10 log genome equivalents/g feces; Santacruz et al., 2010). These results are correlated with the decreased levels of bifidobacteria in babies whose mother gained significant weight during pregnancy (Collado et al., 2008b), that is over and above the pregnancy itself. In terms of allergic disease, Hevia et al. (2016) observed lower levels of *Bifidobacterium* in patients with long-term asthma. The same study showed a dominance of *B. adolescentis* in both short- and long-term asthmatic individuals, in concordance with other previous studies (Stsepetova et al., 2007). This species was only found in infants of allergic mothers, who displayed lower levels of bifidobacteria in their breast milk (Grönlund et al., 2007). One particular Turkish study described a statistical difference between the increased levels of *B. longum* in healthy children (30.3%) when compared to children with allergic disease (11.1%), suggesting that *B. longum* may play a beneficial role in the disease and thus may be useful as a probiotic for the prevention of allergic pathologies (Akay et al., 2014). Different studies have focused on the relationship between the intestinal microbiota and the pathogenesis of irritable bowel syndrome (IBS), showing an altered microbiota related to IBS patients and lower levels in the *Bifidobacterium* genus (Taverniti and Guglielmetti, 2014).

In the elderly gut microbiota, Hopkins and Macfarlane (2002) focused on the impact of *Clostridium difficile*-associated diarrhea, describing a reduction in numbers of bifidobacteria in elderly people suffering the infection compared to a healthy control group. Decreased numbers of bifidobacteria have also been observed in other illnesses such as cystic fibrosis, hepatitis B and both diabetes Types I and II (Wu et al., 2010; Xu et al., 2012; Duytschaever et al., 2013; Murri et al., 2013).

Overall, there is a repetitive trend between lower bifidobacteria and a variety of common disease states, suggesting a role of bifidobacteria in health. However, whether bifidobacteria numbers have any causal relationship to any of these conditions remains unknown.

### Bifidobacteria as Probiotics in Diseases

Numerous health-promoting effects have been ascribed to strains of the *Bifidobacterium* genus based on their use as probiotics in intervention strategies to address many ill health conditions.

In that regard, the capability of bifidobacteria to stimulate the immune system seems to be species specific as shown in a particular study where the effect of infant derived bifidobacteria on the T-helper 1(T_H_1)/T_H_2 balance was examined. Results published by Ménard et al. (2008) demonstrated that *B. bifidum, B. dentium*, and *B. longum* were capable of stimulating systemic and intestinal immunity. It is not surprising that bifidobacteria are so widely used as probiotics in the treatment and prevention of infant disease given their dominance in the infant gut. Their application in pathologies such as allergies, celiac disease, obesity, diarrheas, colic, infections or necrotizing enterocolitis has returned very good results (Di Gioia et al., 2014). They have also been extensively used in adults and elderly, in the treatment of gastrointestinal and respiratory diseases (Biagi et al., 2012; Malaguarnera et al., 2012; Tojo et al., 2014). In an attempt to restore the lipoprotein imbalance found in the blood of children with dyslipidemia, Guardamagna et al. (2014) examined the effects of a probiotic which contained three *Bifidobacterium* strains, selected due to their bile salt hydrolase activity. Their results found a decrease in total cholesterol and low-density lipoprotein cholesterol.

In terms of brain gut disorders, several studies have examined the use of bifidobacteria for their psychobiotic effects in reducing stress, anxiety and depressive like behavior in BALB/c mice. It was concluded that the behavioral effects observed may be partially explained by the differential effects on the immune system, although mechanisms underlying the effects are unknown (Savignac et al., 2014). Furthermore, a particular strain of *B. longum* was shown to have anxiolytic effects in a model of non-infectious colitis through vagal pathways whereby the fermentation products of this species were capable of modifying the excitation of enteric neurons in the gut (Bercik et al., 2011).

Bifidobacteria have also been studied for their ability to specifically localize at tumor sites (Nakamura et al., 2002). Cronin et al. (2010) showed in that *B. breve* UCC 2003 was able to target tumors in athymic MF1 nu/nu mice bearing s.c. B16-F10 murine melanoma tumors, as well as C57 mice bearing s.c. Lewis lung carcinoma tumors. Interestingly, an increasing bacterial load of *B. breve* UCC 2003 was found in the metastatic nodules in the lungs of the B-16 mouse model where oxygen is transferred. Bifidobacteria have also been shown to have an effect on tumor specific T-cell responses in C57BL/6 mice bearing s.c. B16.SIY melanoma growth (Sivan et al., 2015). In this study bifidobacteria were found to have an effect on dendritic cell function and CD8+ T cell responses, reducing tumor cell growth.

*Bifidobacterium* species may have positive effects on human health, while it should be stressed that the increase only in fecal bifidobacteria level cannot in itself a health benefit. However, given the strong association of bifidobacteria to health it may provide a contributory biomarker to disease status lined to some illnesses in the future. Moreover the use of bifidobacteria as delivery vehicles for the administration of therapeutic agents to target tumors due to their anaerobic nature, is a very promising and safe form of therapy for this disease state.

## Conclusion

Bifidobacteria are one of the most abundant genera present in the healthy infant gut and represent a significant portion of the microbiota throughout a healthy adult life, playing an important role in gut homeostasis and health. During late adulthood and within several diseases, the levels of *Bifidobacterium* and its species diversity decrease. Nowadays, their association with health and aging is undeniable, however, the jury is still out on whether it is a “cause” or “effect” type relationship. Given the prevalence of bifidobacteria at various stages of a healthy life and the many health promoting attributes associated with their use, it is undoubted that these bacteria play an important role in human health maintenance and protection and also may in the future provide a very important biomarker for certain diseases.

## Author Contributions

RR, CS, and SA conceived the manuscript. SA and CW drafted the manuscript. CS and RR reviewed the final version of the manuscript. All the authors approved it for publication.

## Conflict of Interest Statement

The authors declare that the research was conducted in the absence of any commercial or financial relationships that could be construed as a potential conflict of interest.
